# Longitudinal Changes in Leadership and Person-Centered Care Over 5 Years in Swedish Nursing Homes

**DOI:** 10.1093/geroni/igab046.1443

**Published:** 2021-12-17

**Authors:** Annica Backman, karin sjögren, Hugo Lövheim, Marie Lindkvist, David Edvardsson

**Affiliations:** 1 Umea University, Umea, Vasterbottens Lan, Sweden; 2 La Trobe University, La Trobe University Melbourne, Victoria, Australia

## Abstract

Nursing home leadership has been described as crucial for person-centred care and psychosocial climate, but longitudinal data are lacking. The significance of manager educational qualifications and operational model of nursing homes for perceived leadership, person-centred care and psychosocial climate also needs further exploration. This study aimed to explore changes in nursing home managers’ leadership, person-centred care and psychosocial climate comparing matched units in a five-year follow-up. Also, to explore changes in leadership characteristics’ and the significance of manager qualifications for perceived leadership, person-centred care and climate. Repeated cross-sectional, valid and reliable, measures of leadership, person-centred care, psychosocial climate and demographic variables were collected from managers and staff n=3605 in 2014 and n=2985 staff in 2019. Descriptive and regression analyses were used. Leadership remained significantly associated to person-centred care in a five-year follow-up, but no changes in strength of associations were seen. Leadership also remained significantly associated to psychosocial climate, with stronger associations at follow-up. Also, certain leadership characteristics significantly increased over time, thus, partly confirms previous findings. It was also shown that a targeted education for managers was significantly associated to person-centred care.

